# Regulation of Flowering Time and Other Developmental Plasticities by 3’ Splicing Factor-Mediated Alternative Splicing in *Arabidopsis thaliana*

**DOI:** 10.3390/plants12193508

**Published:** 2023-10-09

**Authors:** Keh Chien Lee, Young-Cheon Kim, Jeong-Kook Kim, Horim Lee, Jeong Hwan Lee

**Affiliations:** 1 Umeå Plant Science Centre, Department of Forest Genetics and Plant Physiology, Swedish University of Agricultural Sciences, 90183 Umeå, Sweden; keh.chien.lee@slu.se; 2 Division of Life Sciences, Jeonbuk National University, 567 Baekje-daero, Deokjin-gu, Jeonju 54896, Jeollabuk-do, Republic of Korea; yck01000@jbnu.ac.kr; 3 Division of Life Sciences, Korea University, 145 Anam-ro, Seongbuk-gu, Seoul 02841, Republic of Korea; jkkim@korea.ac.kr; 4 Department of Biotechnology, Duksung Women’s University, Seoul 03169, Republic of Korea

**Keywords:** alternative splicing, developmental plasticity, flowering time, root apical meristem, shoot apical meristem, 3’ splicing factors

## Abstract

Plants, as sessile organisms, show a high degree of plasticity in their growth and development and have various strategies to cope with these alterations under continuously changing environments and unfavorable stress conditions. In particular, the floral transition from the vegetative and reproductive phases in the shoot apical meristem (SAM) is one of the most important developmental changes in plants. In addition, meristem regions, such as the SAM and root apical meristem (RAM), which continually generate new lateral organs throughout the plant life cycle, are important sites for developmental plasticity. Recent findings have shown that the prevailing type of alternative splicing (AS) in plants is intron retention (IR) unlike in animals; thus, AS is an important regulatory mechanism conferring plasticity for plant growth and development under various environmental conditions. Although eukaryotes exhibit some similarities in the composition and dynamics of their splicing machinery, plants have differences in the 3’ splicing characteristics governing AS. Here, we summarize recent findings on the roles of 3’ splicing factors and their interacting partners in regulating the flowering time and other developmental plasticities in *Arabidopsis thaliana*.

## 1. Introduction

The splicing process, in which the introns and non-coding regions of pre-messenger RNA (pre-mRNA) are removed and the remaining exons are joined to form a mature messenger RNA (mRNA), is a crucial step in mRNA maturation. A large ribonucleoprotein complex called the spliceosome, which consists of small nuclear ribonucleoproteins (snRNPs) and numerous sets of protein cofactors, mediates this process, contributing to the highly dynamic machinery for proper pre-mRNA splicing [1,2,3]. The spliceosome recognizes the conserved *cis*-elements, including the 5’ splice site (5’ ss), the branch point site (BPS), the polypyrimidine tract (PPT), and the 3’ splice site (3’ ss) of the introns of pre-mRNAs, and brings these sites together to form a lariat intermediate. Furthermore, the adenine-uridine (AU)-rich sequences throughout the whole length of the introns in plants are essential for recognizing the introns unlike in animals [4,5]. Splicing regulation is critical in multiple biological processes, including development, differentiation, and abiotic and biotic stresses. Defects in splicing can lead to genetic disorders and even embryonic lethality [6,7,8]; thus, understanding the molecular mechanism of splicing is essential for understanding gene expression and its role in animal and plant development.

Although the splicing factors involved in constitutive splicing and alternative splicing (AS) are essentially the same, they play different roles in organisms. Constitutive splicing causes the production of a defined protein in which all the exons are included from mature mRNAs, whereas AS redirects primary transcripts into one of two major pathways: (1) towards protein synthesis if the spliced mRNAs consist of exons or (2) towards nonsense-mediated decay (NMD) if the spliced mRNAs carry a premature termination codon (PTC) [9,10]. The AS pathway results in functional diversification and proteome expansion by producing distinct protein isoforms, whereas the NMD pathway controls gene expression by removing nonsense mRNA transcripts. Thus, AS can be regulated by various factors, including *cis*-acting RNA elements, splicing regulatory proteins, and chromatin structure [11,12,13,14].

Although the splicing mechanisms and splicing factors are largely conserved between animal and plant counterparts [10,15], there are some key differences in the 3’ splicing characteristics and regulatory mechanisms governing AS between animals and plants. In plants, uridine (U)-rich sequences toward the 3’ ss is found to be an essential determinant of the splicing efficiency [10,16,17]. For instance, the AU and guanine-cytosine (GC) content of introns and exons are different. Plants exhibit several unique characteristics of AS, including intron retention (IR), alternative 3’ ss selection, and alternative polyadenylation. It has been well documented that IR is more prevalent in plants than in animals and is estimated to account for approximately one third of the alternative splicing events in plant development [10,18]. Plants also exhibit a higher degree of alternative 3’ ss selection and polyadenylation than animal counterparts [19]. This leads to the production of mRNA isoforms with different 3’ untranslated regions (3’ UTRs) that can affect mRNA stability, translation efficiency, and localization.

Plants are sessile organisms that are largely dependent on environmental cues for growth and survival. Accumulating evidence suggests that the AS genes regulate the environmental fitness of plants as a fine-tuning molecular mechanism for adaptation to a changing environment [20]. The molecular mechanisms of AS producing multiple transcripts from a single gene are involved in regulating the gene expression for the flowering time and plant development, mainly through the transcriptional or translational control of alternatively spliced isoforms [21,22,23]. In this review, we summarize the roles of these 3’ splicing factors and their interacting proteins that modulate proper pre-mRNA splicing in regulating flowering time and other developmental plasticities in *Arabidopsis thaliana*.

## 2. Regulating Flowering Time and Other Developmental Plasticities by Alternative Splicing via 3’ Splicing Factors

The 3’ splicing factors and their interacting proteins bind to the 3’ ss of pre-mRNAs to catalyze the splicing reaction that removes the introns and joins the exons together. Several 3’ splicing factors have been implicated in the regulation of flowering time and development in *Arabidopsis thaliana* (Table 1). It is well-known that the AS of key player genes including transcriptional control of MADS-box transcription factor, *FLOWERING LOCUS C* (*FLC*) [24,25], temperature-dependent AS of *FLOWERING LOCUS M* (*FLM*) [26], *FLOWERING CONTROL LOCUS A* (*FCA*) [27]; and the circadian clock genes, including *PSEUDO-RESPONSE REGULATOR 7* (*PRR7*) and *PRR9* [28] are involved in regulating flowering time control. The AS of these genes, modulated by splicing factors, comprising the large spliceosome assembly machinery, is crucial for proper pre-mRNA splicing. Here, we summarize the recent knowledge of the 3′ splicing factors and their interacting partners involved in floral transition, developmental plasticity, and abiotic stress in plants.

### 2.1. Roles of SnRNP-Specific Proteins

**Figure 1:**
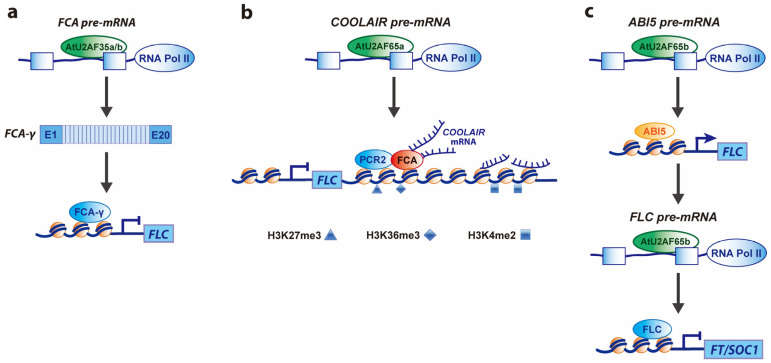
**Regulatory mechanisms of flowering time by *At*U2AF35 and *At*U2AF65.** (**a**) A possible mechanism of flowering by *Arabidopsis* U2 auxiliary factor 35 (*At*U2AF35)-mediated alternative splicing of *FLOWERING CONTROL LOCUS A* (*FCA*) pre-mRNA. Normal levels of *At*U2AF35 produce major functional *FCA-γ* transcripts from *FCA* pre-mRNA, thereby leading to the binding of FCA-γ to *FLOWERING LOCUS C* (*FLC*) locus, which in turn represses flowering under unfavorable conditions. (**b**) A possible mechanism of flowering by *Arabidopsis* U2 auxiliary factor 65a (*At*U2AF65a)-mediated production of *COOLAIR* transcripts. *COOLAIR* long non-coding antisense RNAs expressed from the *FLC* locus are important in regulating *FLC* chromatin silencing and transcriptional repression in nonvernalized plants [25,34,35,36]. *COOLAIR* RNAs are classified into Class I (proximal isoforms) and Class II (distal isoforms) according to the positions where polyadenylation occurs by 3’ end-processing factors [37,38,39]. *At*U2AF65a binds to the *FLC* locus to affect the expression of *COOLAIR* *Class I* and *Class II* RNAs, leading to the binding of *COOLAIR Class I* RNAs to the *FLC* locus, which affects the histone methylation of H3K4me2, H3K36me3, and H3K27me3. In addition, two classes of *COOLAIR* transcripts bind to FCA and recruit PRC2 complex to the *FLC* locus, thereby repressing *FLC* expression. (**c**) A possible mechanism of flowering by *At*U2AF65b-mediated alternative splicing of targets’ pre-mRNAs. Increased expression levels of *AtU2AF65b* induced by abscisic acid (ABA) binds to the pre-mRNA of *ABSCISIC ACID-INSENSITIVE 5* (*ABI5*), thereby leading to binding of ABI5 to *FLC* genomic regions, which in turn represses flowering by increased *FLC* expression [25]. In addition, *At*U2AF65b binds to *FLC* pre-mRNA to affect *FLC* splicing. However, *At*U2AF65b may regulate flowering time in an ABA-independent manner [24]. Solid and T-shaped arrows indicate activation and repression of target genes’ expression, respectively.

Several splicing factors are involved in pre-mRNA splicing and participate in the spliceosome assembly. Several splicing factors involved in 3’ ss and PPT binding have been identified, including two subunits of the U2 auxiliary factor (U2AF65 and U2AF35) [2]. U2AF binds to the PPT between the intron BPS and the 3’ AG dinucleotide intron boundary to recruit U2 SnRNP to the BPS. The U2AF proteins are composed of a 35 kDa subunit of U2AF35, which binds to the 3’ AG boundary, and the larger 65 kDa subunit (U2AF65), binding directly to the PPT upstream of 3’ ss. The binding of 3’ ss by U2AF35 promotes the binding of U2AF65 to PPT sequences and interacts with serine/arginine (SR) proteins [29,30,31]. U2AF65 and U2AF35 also exist as two homologs (*At*U2AF65a and *At*U2AF65b, and *At*U2AF35a and *At*U2AF35b, respectively) in *Arabidopsis* [27,32]. The recent evidence suggests that *At*U2AF35a/b and *At*U2AF65a/b play important roles in regulating flowering time by regulating the AS of key flowering genes (Figure 1, Table 1) [24,25,27]. The *atu2af35a* and *atu2af35b* mutants showed late-flowering phenotypes under both long-day (LD) and short-day (SD) conditions [27]. In addition to the flowering time, *atu2af35* mutants also showed pleiotropic phenotypes, including abnormal leaf morphology, flowers, and silique shape. Furthermore, mutations in *AtU2AF35* altered the expression level of the flowering time gene *FCA* due to altered AS in *FCA* isoforms, suggesting that the increased abnormal *FCA* transcripts could not repress the *FLC* expression (Figure 1a). Loss-of-function mutants of the two isoforms of *AtU2AF65* also reveal their functional roles in floral transition [24,25,33]. *atu2af65a* and *atu2af65b* mutants showed late- and early- flowering phenotypes, respectively, which correlated with altered expression levels of the flowering time genes, including *FLC* and *FLOWERING LOCUS T* (*FT*) in the leaves [24]. In addition, RNA-sequencing (RNA-seq) analysis in the shoot apex regions of wild-type (Col-0), *atu2af65a*, and *atu2af65b* plants revealed that the expression levels or AS patterns of *COOLAIR* long non-coding RNAs (lncRNAs), *EDM2*, or *PP2A-b’ɤ*, the *FLC* upstream regulators, were changed in the shoot apices of *atu2af65a* mutants [24], suggesting that *At*U2AF65a regulated the *FLC* expression through *COOLAIR RNA*-mediated *FLC* repression, and the reduced expression of *EDM2* and *PP2A-b’ɤ* in vernalization-mediated flowering (Figure 1b). Furthermore, *At*U2AF65b is known to be involved in the pre-mRNA splicing of *ABSCISIC ACID-INSENSITIVE 5* (*ABI5*), which encodes an activator of *FLC* in abscisic acid (ABA)-mediated flowering, both because of the reduced *FLC* transcription and IR of *FLC* (Figure 1c) [25]. Double mutations in *AtU2AF65a* and *AtU2AF65b* also result in defective male gametophytes due to impaired pollen tube growth [24]. These results suggested that the two subunits of the *At*U2 auxiliary factor (*At*U2AF65 and *At*U2AF35) affect the flowering time and other plant developmental processes.

**Figure 2:**
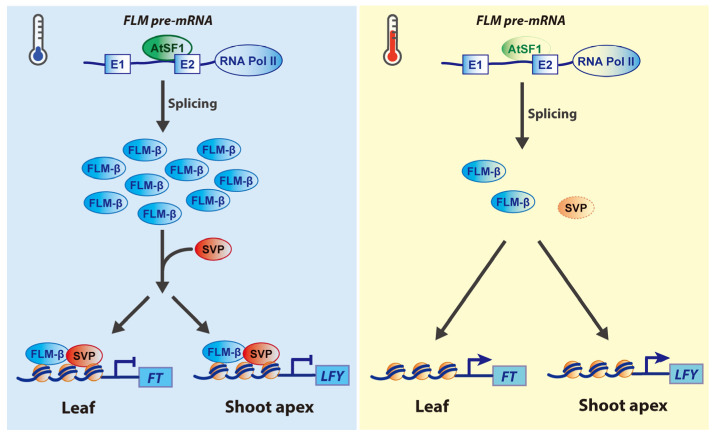
**A regulatory mechanism of temperature-dependent flowering by *At*SF1-mediated alternative splicing of *FLM* pre-mRNAs.** At low ambient temperatures (blue thermometer), *Arabidopsis* splicing factor1 (*At*SF1) strongly binds to the branch point site (BPS) of *FLOWERING LOCUS M* (*FLM*) pre-mRNA intron 1 to produce major functional *FLM-β* transcripts, thereby leading to the formation of the SHORT VEGETATIVE PHASE (SVP)–FLM-β repressor complex, which in turn represses flowering by binding its complex to the genomic regions of the floral activators such as *FLOWERING LOCUS T* (*FT*) and *LEAFY* (*LFY*) in the leaves and shoot apices, respectively [41,42,47]. At warm ambient temperatures (red thermometer), the binding of *At*SF1 to the BPS in intron 1 of *FLM* pre-mRNA is significantly reduced. The lower level of *FLM-β* transcripts and the degradation of SVP results in decreased levels of the SVP–FLM-β complex and thus release the repression of *FT* and *LFY* expression in the leaves and shoot apices, respectively, thereby inducing flowering. Thick and thin lines of *At*SF1 denote strong and weak binding to the BPS in intron 1 of *FLM* pre-mRNA, respectively. Solid and T-shaped arrows indicate activation and repression of target genes’ expression, respectively.

In addition to the interaction between U2AF65 and U2AF35 for the recognition of the 3’ ss, splicing factor 1 (SF1) associated with U2AF65 recognizes the 3’ ss and binds to the BPS of introns [2]. Plant homolog splicing factor 1 (*At*SF1) has also been identified in *Arabidopsis* using forward and reverse genetic approaches [32,40]. A recent study showed that *At*SF1 differentially binds to the BPS of different introns in *FLM* pre-mRNA in a temperature-dependent manner to regulate the production of the major functional *FLM-β* transcripts, thereby eventually affecting the temperature-responsive flowering (Figure 2, Table 1) [41]. Lee et al. [42] reported that mutant lines harboring a deletion of the RNA recognition motif (RRM) domain of *At*SF1 did not recover from the defect in the flowering time, suggesting that the RRM domain of *At*SF1 is important for regulating the flowering time. Genetic interactions and chromatin immunoprecipitation (ChIP) analyses revealed that the *AtSF1-FLM* module regulates temperature-dependent flowering by regulating the *FLOWERING LOCUS T* (*FT*) and *LEAFY* (*LFY*) expression in the leaf and shoot apex regions, respectively [41]. Furthermore, mutations in *AtSF1* result in developmental abnormalities, including plastochron length, dwarfism, and hypersensitivity to abscisic acid during seed germination, heat stress, and chloroplast development under cold stress [32,41,42,43], suggesting that *At*SF1 is essential for various developmental processes and abiotic stresses.

SF1, U2AF35, and U2AF65 function at the early stage of pre-mRNA splicing, during which it binds the BPS, the PPT, and the 3’ ss of the intron of the pre-mRNAs [2,29]. However, BPS and PPT are less well-conserved in plant species [44,45,46]. Furthermore, the *atu2af65a* and *atu2af65b* mutants showed opposite flowering times [24], and the expression and AS of more genes were affected specifically by the *atu2af65a* mutation compared to the *atu2af65b* mutation [24]. Thus, further analysis of the interaction between specific 3’ splicing factors (*At*SF1, *At*U2AF35, and *At*U2AF65) and RNA sequences is required.

### 2.2. Roles of SR Proteins

The first known plant splicing factors that regulate pre-mRNA splicing and spliceosome assembly were the SR family proteins [48]. They affect 5’ or 3’ ss selection in a concentration- and phosphorylation-dependent fashion, thereby contributing to the AS process in a tissue-specific, developmentally regulated, and stress-responsive manner [1,49]. Members of the SR protein family are characterized by their ability to interact simultaneously with RNA and proteins via one or two N-terminal RNA recognition motifs (RRM) and a C-terminal arginine- and serine-rich (RS) domain. The *Arabidopsis* genome encodes 18 SR proteins that represent six different subfamilies, three of which (SR, RSZ, and SC) are orthologous to the animal SR proteins, whereas the other three are plant-specific and have structural features that are not found in the animal kingdom [1]. These include SR (*At*SR30, *At*SR34, *At*SR34a, and *At*SR34b), RSZ (*At*RSZ21, *At*RSZ22, and *At*RSZ22a), SC (*At*SC35), SCL (*At*SCL28, *At*SCL30, *At*SCL30a, and *At*SCL33), RS2Z (*At*RS2Z32 and *At*RS2Z33), and RS (*At*SR31, *At*SR31a, *At*SR40, and *At*SR41) subfamilies. For example, the members of the SCL subfamily, which are structurally related to the SC subfamily, have a unique N-terminal domain rich in charged amino acids. RS2Z members, resembling the RSZ subfamily, possess an additional zinc knuckle and a serine- and proline-rich acidic carboxyl-terminal domain [50]. Although some studies have suggested the functional redundancy of the plant SR proteins [51], the diversification or redundancy of their functions is still not completely understood.

**Figure 3:**
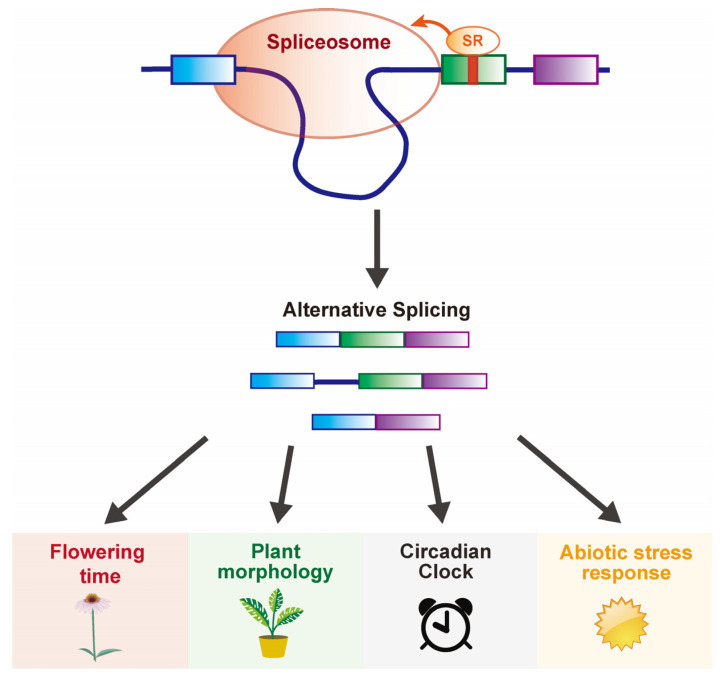
**Model of SR proteins in the regulating flowering time and other developmental plasticity.** SR proteins as components of spliceosome mediate the pre-mRNA splicing of flowering time, plant morphology, circadian clock, and abiotic stress responses-related genes at the post-transcriptional level.

Among these SR proteins, *At*SR40, *At*RSZ22, *At*SCL30, and others (*At*SR45 and *At*RSZ22a) interact with the cold-induced LAMMER KINASE AME3, and their loss-of-function mutants exhibit cold sensitivity [52], suggesting that these SR proteins are involved in abiotic stresses such as cold acclimation and acquisition of freezing tolerance by regulating their phosphorylation status. This notion is supported by the observation that the kinase AFC2 autophosphorylates and phosphorylates four plant SR proteins (SRZ21, SRZ22, SRp33, and SR45) and that the interaction between AFC2 and SR33 is altered by the phosphorylation status of these proteins [53]. Yan et al. [51] also found that *At*SC35 and other *At*SCL proteins affect various developmental processes, such as the leaf and root morphology, flowering time, and silique phyllotaxy (Figure 3, Table 1). For instance, quintuple mutants of *AtSC35* and four *AtSCLs* caused pleiotropic changes in the plant morphology and development, including serrated leaves, delayed flowering, shorter roots, and abnormal siliques. These phenotypes were affected by changes in the AS patterns of 213 genes and the transcription of a subset of genes. In particular, the splicing of *FLC* intron 1 and its transcription are significantly altered in the quintuple mutants. These developmental defects may be due to the depletion of *At*SC35 and other SCL proteins that interact with NRPB4, a specific subunit of RNA polymerase II.

*AtSR45* mutants exhibited pleiotropic defects, including a delayed flowering, abnormal leaf morphology, altered petal and stamen numbers, reduced root growth, and hypersensitivity to glucose and ABA (Figure 3, Table 1) [12,46]. The *atsr45* mutants also showed altered splicing patterns of several SR genes, including changes in the AS of *AtSR30* pre-mRNA [46]. *At*SR45 is also involved in the AS of the circadian clock gene *circadian clock associated 1* (*CCA1*). These results suggest that *At*SR45 regulates developmental plasticity without functional redundancy.

Altered splicing patterns in the circadian clock genes, including *PSEUDO-RESPONSE REGULATOR 7* (*PRR7*) and *PRR9*, have also been reported in *snw/ski interacting protein* (*skip*)*-1* mutant, a splicing factor that interacts physically with *At*SR45 [54], suggesting that SKIP is important for temperature compensation in the circadian clock (Figure 3, Table 1) [55]. Interestingly, this mutant showed an early flowering phenotype under different photoperiods and temperatures [54]. Genetic and molecular analyses have shown that SKIP regulates the flowering time by regulating the pre-mRNA AS of a component of chromatin remodeling, *SERRATED LEAVES AND EARLY FLOWERING* (*SEF*), which eventually affects the H2A.Z enrichment at *FLC*, *MADS AFFECTING FLOWERING 4* (*MAF4*), and *MAF5* [56].

The accumulation of the U1, U2, U4, and U5 small nuclear RNAs (snRNAs) in the spliceosome core complex is facilitated by another spliceosomal core component [57]. Sm protein E1 (SmE1), which is also reported as PORCUPINE (PCP) [58], is an example of such a component that has been demonstrated to be essential for regulating the flowering time and responding to abiotic stress (Table 1) [59]. In *sme1* mutants, RNA-seq analysis showed that the *FLC* expression was severely reduced, which correlated with the early flowering phenotype. In addition, RNA expression analyses revealed that the transcripts of *COOLAIR* class I isoforms accumulated, whereas those of the *COOLAIR* class II isoforms were reduced in *sme1* mutants. These results suggest that SME1 affects the pre-mRNA splicing of *COOLAIR*, thereby leading to the accumulation of *FLC* transcripts.

Several recent studies have revealed that the plant SR proteins affect various developmental plasticities, including the flowering time, plant morphology, and abiotic stress. Considering the large number of SR proteins, distinct structural features in plant-specific subfamilies, and the interactions between them and other spliceosomal proteins [48], a single-gene knockout approach is unsuitable for analyzing their function. Thus, new technologies, such as clustered regularly interspaced short palindromic repeats/CRISPR-associated nuclease9 (CRISPR/Cas9)-mediated genome editing, RNA immunoprecipitation sequencing (RIP-seq), and cross-linking and immunoprecipitation sequencing (CLIP-seq), enable the functional analysis of the plant SR protein family in regulating plant developmental plasticity [60].

## 3. Alternative Splicing-Mediated Developmental Plasticity in Meristems

Developmental plasticity is a critical process in plants because of their sessile properties and determines most developmental changes after embryogenesis during the entire lifespan. Therefore, the plastic properties of plants are required for their adaptive growth and development in response to unfavorable environmental conditions. In plants, primary meristems, including shoot apical meristems (SAMs) and root apical meristems (RAMs), contain undifferentiated stem cells and differentiating cells for lateral organ formation [61]. Thus, the meristem region, which continuously generates new lateral organs under various environmental conditions, is an important site for developmental plasticity, where the underlying molecular mechanisms, such as AS, provide diversity and fine-tune gene expression. In particular, previous studies have shown that small fluctuations in the temperature, such as ambient temperature, directly influence the AS processes and that these changes affect the downstream genes associated with adaptation for plant development in response to changing temperatures [33,62].

### 3.1. SAM Development via Alternative Splicing

**Figure 4:**
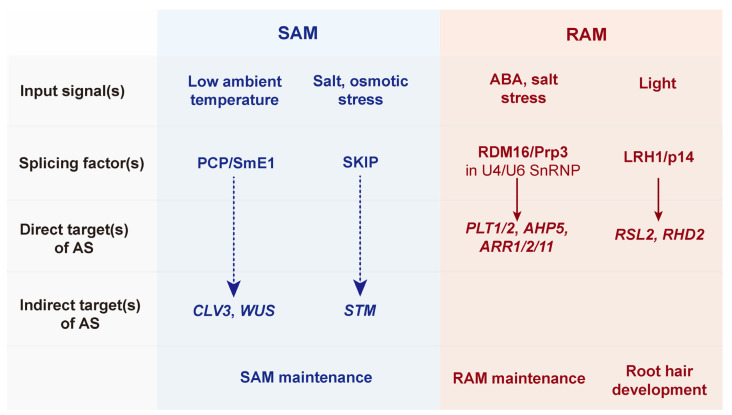
**A scheme of developmental plasticity in apical meristems by alternative splicing.** The 3’ splicing factors in primary apical meristems [shoot apical meristem (SAM) and root apical meristem (RAM)] are associated with various environmental and abiotic stress signals. *ROOT HAIR SIX-LIKE2* (*RSL2*) and *ROOT HAIR DEFECTIVE2* (*RHD2*) are directly alternatively spliced by RNA-directed DNA METHYLATION (RDM16)/Pre-mRNA-splicing factor 3 (Prp3) and LIGHT-SENSITIVE ROOT-HAIR DEVELOPMENT 1 (LRH1)/p14 (solid arrows), respectively, for root growth and development. In contrast, SAM maintenance via CLAVATA3 (CLV3)-WUSCHEL (WUS) negative feedback and *SHOOT MERISTEMLESS* (*STM*) is indirectly (dashed arrows) regulated by 3’ splicing factors such as PORCUPINE (PCP)/SmE1 and SNW/SKI INTERACTING PROTEIN (SKIP).

Recent findings revealed the putative splicing regulator *PCP*, which is related to temperature-sensitive AS with SAM maintenance through a complex regulatory network (Figure 4, Table 1) [58]. In that study, the expression of *PCP* was shown to be down-regulated by elevated temperature fluctuations between 16, 23, and 27 °C using RNA-seq analysis. The *pcp-1* mutant showed severe defects in plant growth, such as the failure of SAM maintenance and arrest of root growth at a low ambient temperature (16 °C), whereas the *pcp-1* mutant grew similarly compared to wild-type plants at a normal temperature (23 °C). Interestingly, the *PCP* gene was shown to generate two transcript variants, *PCP-α* and *PCP-β*, via AS, and only the *PCP-α* isoform acted as an active form for appropriate plant growth at low ambient temperatures. Although the misregulation of the SAM maintenance genes, such as *WUSCHEL* (*WUS*) and *CLAVATA3* (*CLV3*), was found in *pcp-1* mutants at a low ambient temperature (16 °C), but not at 23 °C, neither the *WUS* nor *CLV3* promoter-driven *PCP* expression rescues the *pcp-1* mutant phenotypes, suggesting that the effect of *PCP* associated with temperature-sensitive AS indirectly affects SAM maintenance via regulating *WUS* and *CLV3*.

Intriguingly, *PCP* has also recently been characterized as *SmE1* [59], which encodes a component of the Sm complex that forms a heptameric ring structure around snRNAs [63] and interacts with the Sm-like (LSM) 2-8 complex that regulates the efficiency of constitutive splicing and AS according to the changes in environmental conditions (Figure 4, Table 1) [64,65]. Huertas et al. [59] found that the accumulation of the U1, U2, U4, and U5 snRNAs was reduced, and the alteration of splicing events, including IR, increased at a genome-wide level in *sme1* mutants. Furthermore, the expression of *SmE1* was increased at a low temperature (4 °C), indicating that SmE1 regulates the spliceosome activity depending on the environmental conditions. Interestingly, changes in the splicing events correlated with the developmental defects, such as smaller rosette leaves, early flowering, and short root growth, exhibited in *sme1* mutants at a normal temperature (20 °C), suggesting that SmE1 regulates plant development via splicing activity. Although these two reports showed inconsistent growth phenotypes at normal temperatures owing to the different experimental conditions [58,59], they provide new insights into the developmental plasticity in the SAM via temperature-mediated AS.

In addition to the negative feedback loop of the CLV3-WUS pathway [66], SHOOT MERISTEMLESS (STM) synergistically plays an important role in the regulating the shoot meristem initiation and maintenance (Figure 4, Table 1) [67,68]. STM is also known to maintain the SAM activity through protein-protein interactions that modulate transcriptional regulation and intercellular trafficking [69,70]. Recently, SKIP, a bifunctional factor (splicing factor and transcriptional regulator), was found to interact with STM to regulate the target gene expression for SAM formation [71]. For example, both loss-of-function and CRISPR-Cas9-mediated *skip* mutants show severe developmental defects, such as failed shoot meristem formation, which was also observed in loss-of-function *stm* mutants, suggesting that *SKIP*, like *STM*, is required for SAM initiation and maintenance. In addition, SKIP-STM interactions have been shown to regulate downstream target genes, such as *STM*, *Knotted-1-like 1* (*KNAT1*), *CLV3,* and *GA2-oxidase 1* (*GA2OX1*), by binding to their promoters, supporting the transcriptional function of the SKIP-STM heterodimeric complex in SAM development. Interestingly, in plants, SKIP has previously been reported to interact with spliceosome components and to function in the circadian clock and salt stress responses by regulating AS [54,72], suggesting a splicing function via SKIP in STM-mediated plastic shoot meristem maintenance.

Several recent studies have raised the possibility of AS-mediated plastic development in SAM. However, because the splicing defects of the essential meristem maintenance genes such as *CLV3*, *WUS,* and *STM* are insignificant [58,71], the link between the AS events and the key mechanisms of SAM maintenance for plastic development will be further investigated.

### 3.2. RAM Development via Alternative Splicing

AS events have also been found to modulate the plastic development in root meristems (Figure 4, Table 1). For example, the loss-of-function mutation of *RNA-directed DNA METHYLATION 16* (*RDM16*), which encodes a component of the U4/U6 snRNP complex involved in pre-mRNA splicing, increased the overall alterations in AS events in RNA-seq analysis and showed a short root phenotype [73,74]. In the *rdm16-4* mutant, a truncated RDM16 protein lacking the DUF1115 domain affected the AS of the root stem cell maintenance genes, such as *PLETHORA1* (*PLT1*) and *PLT2*, and the cytokinin signaling genes, such as *ARABIDOPSIS HISTIDINE PHOSPHOTRANSFER PROTEIN5* (*AHP5*), *ARABIDOPSIS RESPONSE REGULATOR1* (*ARR1*), *ARR2,* and *ARR11*, thereby leading to a disordered stem cell niche and reduced cytokinin response during root growth, respectively [74]. RDM16 is a homolog of the yeast pre-mRNA splicing factor 3 (Prp3) protein [75], which is highly conserved in eukaryotes and contains a DUF1115 domain at its C-terminus [76]. Since RDM16 proteins contribute to splicing events through DUF1115-mediated interactions with U4/U6 di-snRNA fragments in the spliceosome complex [77], and the *rdm16* mutant is hypersensitive to salt and ABA with morphological defects [73], these results suggest that RMD16-mediated AS plays an important role in root growth and plastic development.

The formation of root hairs from epidermal cells expands the surface area of the root to absorb water and nutrients from the soil. Therefore, the dynamic morphogenesis of the root system, including root hairs, is important for plants that encounter various soil and environmental conditions [78,79]. A recent study showed that root hair formation, which is dynamically controlled by various environmental cues, is involved in regulating AS (Figure 4, Table 1) [80]. They isolated a novel recessive mutant, *light-sensitive root-hair development 1* (*lrh1*), which exhibited enhanced root hair formation in response to light, whereas primary root elongation was inhibited. Interestingly, *LRH1* is known to encode the p14 protein, a putative component of the SPLICING FACTOR 3b (SF3b) complex, for pre-mRNA splicing [2]. In addition, the SF3b subunit SF3b155 simultaneously binds to both p14 and U2AF65 to form an interaction network with U2 snRNA/pre-mRNA at the branch point for proper splicing [81]. RNA-seq analysis revealed genome-wide alterations associated with AS and the expression of genes related to root hair development in *lrh1* mutants [80]. Moreover, since treatment with the splicing inhibitor pladienolide B (PB) increased root hair formation, similar to the *lrh1* mutant phenotype, these results suggest a possible link between AS and root growth for developmental plasticity in plants. However, as the genes involved in root hair development are not alternatively spliced in *lrh1* mutants, a more direct mechanism by which AS affects root hair development should be further investigated.

The studies demonstrate the involvement of AS in the development of SAM and RAM (Figure 4, Table 1). Since it is also known that AS is directly regulated by various abiotic stresses [82], this suggests that AS plays a crucial role in the plasticity of postembryonic development in meristems depending on unfavorable environmental conditions.

## 4. Conclusions and Future Perspectives

Numerous studies have shown that the AS of the target pre-mRNAs regulated by splicing or splicing-related factors is a central mechanism of the plant growth, development, and abiotic stress responses, ensuring physiological and phenotypic plasticity and mediating the integration of various environmental cues. In this review, we focused on discussing the role of the 3’ splicing factors and their interacting partners in regulating the flowering time and other developmental plasticities, and the selected associated factors of the splicing machinery in the primary meristems.

Although the mechanisms and factors underlying AS are largely conserved among eukaryotes, several plant-specific AS properties exist, including the less conserved *cis*-elements, intron retention (IR), alternative 3’ ss selection, and alternative polyadenylation [10,15,44,45,46,48]. In addition, two copies of the U2 auxiliary factors and plant-specific SR proteins are present in plants [24,27]. Thus, understanding the precise role of AS splicing factors in regulating the plant growth, development, and plant stress responses requires the identification of the direct pre-mRNA targets. The state-of-the-art techniques such as RIP-seq and CLIP-seq will help to identify the in vivo targets as well as the consensus RNA sequences recognized by these splicing factors. Furthermore, identifying new splicing factors and their target pre-mRNAs will expand our understanding of how AS controls plant developmental plasticity. Lastly, recent findings in crop species also show that AS isoforms play multiple roles in plant responses by integrating developmental and environmental signals [23]. Therefore, the study of AS mediated by splicing factors in the model plant *Arabidopsis* and crops provides the strategies to improve the plant productivity under unfavorable conditions.

## Figures and Tables

**Table 1:** The 3’ splicing factors and their interactors involved in flowering time and other developmental plasticities.

Classification	Gene Name	Function
Spliceosome components	*U2 auxiliary factor 35* (*AtU2AF35a* and *AtU2AF35b*)	Flowering time; leaf morphology; flower and silique shape
	*U2 auxiliary factor 65a* (*AtU2AF65a*)	Flowering time; pollen tube growth
	*U2 auxiliary factor 65b* (*AtU2AF65b*)	ABA-dependent and -independent flowering time; pollen tube growth
	*Splicing factor 1* (*AtSF1*)	Temperature-dependent flowering time; vegetative growth; ABA response during seed germination; heat stress; chloroplast development under cold stress
	*RNA-directed DNA METHYLATION 16* (*RDM16*)	Root apical meristem development; ABA and salt responses
	*LIGHT-SENSITIVE ROOT-HAIR DEVELOPMENT 1* (*LRH1*)/*p14*	Root hair development
	*SNW/SKI INTERACTING PROTEIN* (*SKIP*)	Flowering time; circadian clock; salt stress
	*PORCUPINE* (*PCP*)/*SmE1*	Flowering time; shoot apical meristem development; leaf morphology; root growth
SR proteins	*SERINE/ARGININE RICH PROTEIN SPLICING FACTOR 40* (*AtSR40*)	Cold acclimation; acquisition of freezing tolerance
	*SERINE/ARGININE RICH PROTEIN SPLICING FACTOR 45* (*AtSR45*)	Flowering time; leaf and root morphology; flower development; cold acclimation; acquisition of freezing tolerance; ABA and glucose responses
	*RS-CONTAINING ZINC FINGER PROTEIN 22* (*AtRSZ22*)	Cold acclimation; acquisition of freezing tolerance
	*SC35-LIKE SPLICING FACTOR* (*AtSC35*)	Flowering time; leaf and root morphology; silique phyllotaxy
	*SC35-LIKE SPLICING FACTOR 30* (*AtSCL30*)	Flowering time; leaf and root morphology; silique phyllotaxy; cold acclimation; acquisition of freezing tolerance

## Data Availability

The data is contained within the manuscript.
